# The First Report of a Methicillin-Resistant *Staphylococcus aureus* Isolate Harboring Type IV SCC*mec* in Thailand

**DOI:** 10.3390/pathogens10040430

**Published:** 2021-04-04

**Authors:** Wichai Santimaleeworagun, Praewdow Preechachuawong, Wandee Samret, Tossawan Jitwasinkul

**Affiliations:** 1 Department of Pharmacy, Faculty of Pharmacy, Silpakorn University, Nakhon Pathom 73000, Thailand; 2 Pharmaceutical Initiative for Resistant Bacteria and Infectious Diseases Working Group [PIRBIG], Faculty of Pharmacy, Silpakorn University, Nakhon Pathom 73000, Thailand; 3 Microbiology Unit, Hua Hin Hospital, Prachuab Kiri Khan 77110, Thailand; santimaleeworag_w@su.ac.th; 4 Pharmacy Unit, Hua Hin Hospital, Prachuab Kiri Khan 77110, Thailand; wandeehuahin@gmail.com; 5 Department of Biopharmacy, Faculty of Pharmacy, Silpakorn University, Nakhon Pathom 73000, Thailand; tosskiji@gmail.com; 6 Bioactives from Natural Resources Research Collaboration for Excellence in Pharmaceutical Sciences (BNEP), Faculty of Pharmacy, Silpakorn University, Nakhon Pathom 73000, Thailand

**Keywords:** CA-MRSA, *S. aureus*, *mec* IV, whole-genome sequencing

## Abstract

Methicillin-resistant *Staphylococcus aureus* (MRSA) is mostly found in Thailand in the hospital as a nosocomial pathogen. This study aimed to report the genetic characterization of a clinical community-acquired MRSA (CA-MRSA) isolate collected from hospitalized patients in Thailand. Among 26 MRSA isolates, *S. aureus* no. S17 preliminarily displayed the presence of a staphylococcal cassette chromosome mec (SCC*mec*) type IV pattern. The bacterial genomic DNA was subjected to whole-genome sequencing. Panton–Valentine leukocidin (PVL) production, virulence toxins, and antibiotic resistance genes were identified, and multi-locus sequence typing (MLST) and spa typing were performed. The strain was matched by sequence to MLST type 2885 and spa type t13880. This strain carried type IV SCC*mec* with no PVL production. Five acquired antimicrobial resistance genes, namely *blaZ*, *mecA*, *Inu(A)*, *tet(K)*, and *dfrG* conferring resistance to β-lactams, lincosamides, tetracycline, and trimethoprim, were identified. The detected toxins were exfoliative toxin A, gamma-hemolysin, leukocidin D, and leukocidin E. Moreover, there were differences in seven regions in CR-MRSA no. S17 compared to CA-MRSA type 300. In summary, we have reported the ST2885-SCC*mec* IV CA-MRSA clinical strain in Thailand for the first time, highlighting the problem of methicillin resistance in community settings and the consideration in choosing appropriate antibiotic therapy.

## 1. Introduction

*Staphylococcus aureus* is presently one of the most important Gram-positive bacteria causing skin/soft tissue, bloodstream, bone/joint infections, pneumonia and endocarditis, in addition to being a rare cause of urinary tract infection and oral infection, in both community and healthcare settings [1,2,3,4]. Since the introduction of β-lactam antibiotics, methicillin-resistant *Staphylococcus aureus* (MRSA) isolates have been identified. MRSA infection increases the risk of death, the duration of hospitalization, and medical costs [5,6].

*S. aureus* becomes resistant to methicillin after acquiring *mecA*, which is usually carried on the staphylococcal cassette chromosome mec (SCC*mec*) as a mobile genetic element. *mecA* can produce a variant of penicillin-binding protein 2, which alters the affinity for β-lactam antibiotics. At present, 13 SCC*mec* elements have been identified. Of these, SCC*mec* types IV and V are carried by CA-MRSA strains [7].

MRSA strains are usually the causes of hospital-acquired MRSA (HA-MRSA) infections; however, MRSA infections in the community (community-acquired MRSA (CA-MRSA)) have been increasingly reported [8]. Regarding the prevalence of CA-MRSA in previous reports, most MRSA infections in Thailand were hospital-associated, whereas CA-MRSA infection was not evident. Only Lulitanond et al. characterized 14 MRSA isolates from infectious outpatients as community onset. Almost all MRSA isolates carried SCC*mec* type III, whereas only one isolate belonged to SCC*mec* type IX. Mekviwattanawong et al. suspected CA-MRSA infection in two patients presenting with three MRSA isolates within 72 h after hospitalization. Unfortunately, the molecular type of these isolates of MRSA was not determined [9]. The characteristics of CA-MRSA strains were not fully investigated, and the reports of the SCC*mec* types of MRSA are limited. In this study, we performed the first genetic characterization of a clinical CA-MRSA isolate collected from patients in Thailand.

## 2. Materials and Methods

### 2.1. Bacterial Isolate

In our previous studies, we collected 26 MRSA isolates from the clinical specimens of inpatients admitted to Hua Hin Hospital, a 400-bed general hospital in western Thailand, between January 2015 and December 2016 [10]. In our previous findings, three different spa types were identified among 16 isolates, and the proportion of strains assigned to spa type t045 was 75%. The remaining spa types were t439 (18.7%) and t13880 (6.3%). A *S. aureus* strain S17 isolate belonging to spa type t13880 exhibited a SCC*mec* type IV pattern. This isolate was stored in trypticase soy broth plus 15% glycerol at −80 °C for future molecular characterization and antimicrobial susceptibility testing.

### 2.2. Phenotypic Study of Antimicrobial Susceptibility Testing

*S. aureus* strain S17 (CA-MRSA no. 17) was phenotypically confirmed to be resistant to cefoxitin using the Kirby–Bauer method based on Clinical and Laboratory Standards Institute (CLSI) guidelines [11]. CA-MRSA no. 17 was subjected to antimicrobial susceptibility testing for vancomycin, linezolid, clindamycin, fusidic acid, erythromycin, and trimethoprim/sulfamethoxazole using E-test methods (Liofilchem, Teramo, Italy). The broth microdilution method was based on CLSI guidelines [11].

### 2.3. The SCCmec Pattern

The genomic DNA of CA-MRSA no. 17 isolate was extracted using a commercial kit (Thermo Fisher Scientific, Waltham, MA, USA) with the inclusion of lysostaphin (Sigma-Aldrich, St. Louis, MO, USA).

For multiplex PCR to determine the SCC*mec* type, the SCC*mec* M-PCR typing assay contained nine pairs of primers including the specific primers for SCC*mec* subtypes I, II, III, IVa, IVb, IVc, IVd, and V and the primers for *mecA*. The PCR conditions followed previously described protocols [12]. The PCR amplicons were visualized using a UV light box after electrophoresis on a 2% agarose gel.

### 2.4. Whole-Genome Sequencing and Data Analysis

The representative MRSA isolate was further subjected to whole-genome sequencing. Bacterial genomic DNA was sequenced using the Illumina Miseq platform. The raw whole genome was de novo assembled using the Assembler 1.2 program (https://cge.cbs.dtu.dk/services/Assembler/ accessed on 14 November 2020) [13]. We confirmed bacterial species by submitting the whole-genome sequence to KmerFinder version 3.0.2 (https://cge.cbs.dtu.dk/services/KmerFinder/history.php accessed on 14 November 2020) [14].

In the present study, the representative isolates were further classified by clonal relationship based on multi-locus sequence typing (MLST) and then analyzed using MLST 2.0 version 2.0.4 (https://cge.cbs.dtu.dk/services/MLST/ accessed on 14 November 2020) [13] and spa typing via spaTyper version 1.0 (https://cge.cbs.dtu.dk/services/spatyper/ accessed on 14 November 2020) [15]. The presence of Panton–Valentine leukocidin (*lukF* and *lukS*) as a virulence toxin was also analyzed using VirulenceFinder-2.0 (https://cge.cbs.dtu.dk/services/VirulenceFinder/ accessed on 14 November 2020).

The antibiotic resistance genes obtained via chromosomal mutation and acquired resistance were identified by the Center of Genomic Epidemiology via ResFinder 3.2 (https://cge.cbs.dtu.dk/services/ResFinder/ accessed on 14 November 2020). The target genes assessed for chromosomal point mutations included *pbp4* (high-level and broad-spectrum resistance to the entire class of β-lactam drugs), *fusA* (high-level resistance to fusidic acid), *gyrA* (encoding DNA gyrase for fluoroquinolone resistance), *grlA*/*grlB* (encoding topoisomerase IV for fluoroquinolone resistance), *ileS* (conferring mupirocin resistance), *dfrB* (trimethoprim resistance), *rpoB* (conferring resistance to rifampin), and 23S (23S rRNA subunit for linezolid resistance). In *S. aureus*, acquired resistance genes included genes related to aminoglycoside, β-lactam, oxazolidinone, quinolone, macrolide, sulfonamide, fusidic acid, glycopeptide, rifampicin, colistin, fosfomycin, trimethoprim, and tetracycline resistance [16].

The CGView Server Stothard Research Group is a comparative genomics tool for circular genomes that allows sequence feature information to be visualized in the context of sequence analysis results [17].

### 2.5. Genome Sequence Data in Sequence Read Archive (SRA)

The raw reads and assembled contigs of CA-MRSA no. 17 was deposited in SRA (NCBI) under the BioProject ID: PRJNA692064 and BioSample accession number SAMN17313309.

## 3. Results

Phenotypically, the minimum inhibitory concentrations for vancomycin, linezolid, clindamycin, fusidic acid, erythromycin, doxycycline, and trimethoprim/sulfamethoxazole (TMP-SMX) in the strain were 0.38, 1.5, 0.19, 0.13, 0.25, 8, and 0.38 μg/mL, respectively.

Our studied CA-MRSA no. 17 isolate was confirmed to be *S. aureus* by submitting the whole-genome sequence to KmerFinder. In MLST analysis, this strain was matched to sequence type 2885. We also confirmed the spa type as t13880 via spaTyper version 1.0. In multiplex PCR assay, CA-MRSA no. S17 carried type IV SCC*mec*. The strain was confirmed to lack PVL.

We used the ResFinder 3.2 online tool to identify antibiotic resistance genes in our whole-genome assembly. There was a wide distribution of antibiotic resistance genes consisting of *blaZ*/*mecA*, *Inu(A)*, *tet(K)*, and *dfrG* conferring resistance to β-lactams, macrolides, tetracycline, and TMP, respectively (Table 1). There were no mutations found in *pbp4*, *fusA*, *gyrA*, *grlA*, *grlB*, *ileS*, *dfrB*, *rpoB*, and 23S. Overall, the antibiotic resistance genotypes exhibited concordance with the observed phenotypic susceptibility testing data. Concerning the presence of toxin genes analyzed via VirulenceFinder-2.0, exfoliative toxin A, gamma-hemolysin, leukocidin D, and leukocidin E were identified (Table 2).

**Figure 1:**
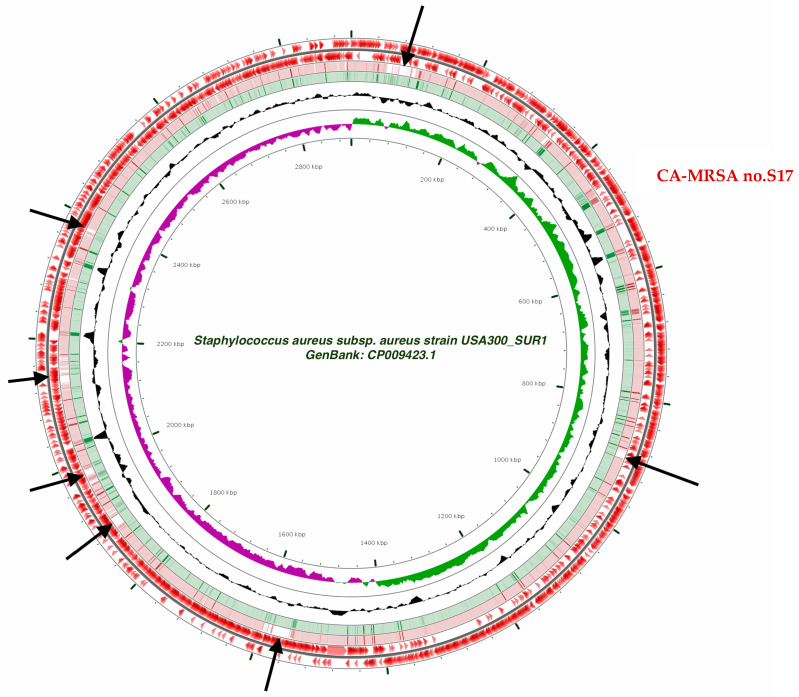
CA-MRSA no. S17 circular genome map (2,904,778 base pairs) compared to CA-MRSA type USA300. Black arrows denote the positions of sequence differences between CA-MRSA type USA300 and CA-MRSA no. S17.

We compared the circular genome sequence (2,904,778 base pairs) of the studied strain to that of CA-MRSA type USA300, finding differences in seven regions (Figure 1).

## 4. Discussion

MRSA was initially recognized as a nosocomial pathogen. The bacterium was not isolated in the community setting until 1961. However, the first definite case of CA-MRSA was reported in 1993 in Western Australia. Subsequently, CA-MRSA was identified in the United States in children who died of sepsis or necrotizing pneumonia between 1997 and 1999. Currently, CA-MRSA strain USA300 is a major cause of CA-MRSA infections in the United States and Canada [7].

As previously described, CA-MRSA has been particularly recognized in the United States over the last two decades. The burden of CA-MRSA infection has shifted the empirical antimicrobial therapy for skin and soft-tissue infection [18]. The United States Centers for Disease Control and Prevention gave the standard definition in 2005 that a CA-MRSA infection is any MRSA infection diagnosed in an outpatient or patient admitted to a hospital within the preceding 48 h [7]. Otherwise, MRSA infections are considered to be HA-MRSA. Our CA-MRSA strain was isolated from a hospitalized patient. However, HA-MRSA strains are also present in the community, especially in patients who required acute care hospital for ≥2 days within the previous 90 days, whereas CA-MRSA isolates can be transmissible in the hospital [18]. Nosocomial transmission and hospital outbreaks of CA-MRSA have been reported in certain countries [19,20]. Thus, the CA-MRSA isolate obtained in this report is the first reported strain in Thailand despite being isolated from a hospitalized patient.

PVL, a cytotoxic toxin, is associated with severe pneumonia and skin/soft-tissue infections caused by *S. aureus*. PVL is encoded by *lukS-PV* and *lukF-PV*. PVL mainly destroys polymorphonuclear leukocytes, monocytes, and macrophages, leading to cell destruction and the release of pro-inflammatory cytokines and nuclear factor-kappa B [21]. PVL production results in necrotizing damage such as lung injury and deep-seated skin and soft-tissue infections [22,23]. PVL-positive *S. aureus* is present in most patients with community-acquired necrotizing pneumonia, among whom the mortality rate ranges between 40%–60% [24].

The prevalence of PVL-positive *S. aureus* among methicillin-resistant isolates varies. Moran et al. enrolled adult patients with purulent skin and soft-tissue infections across 11 university-affiliated emergency departments in the United States in 2004. Of 214 MRSA isolates characterized as SCC*mec* type IV, *pvl* was present in 213 isolates [25]. Similarly, Nakaminami et al. found that all CA-MRSA isolates with SCC*mec* type IV were PVL- positive [26]. However, in other studies, the prevalence of PVL-positive CA-MRSA was the same as that of PVL-negative CA-MRSA [27,28]. Thus, the absence of *pvl* in our CA-MRSA isolate might be reasonable.

Although our CA-MRSA isolate lacked *lukS-PV* and *lukF-PV*, other virulence factors such as exfoliative toxin, hemolysin, and leukocidin were present. Our results support that CA-MRSA strains usually produce hemolysins, leukocidin, or exfoliative toxins, whereas HA-MRSA usually does not produce such toxins. Moreover, CA-MRSA strains also produce β-lactamase and hyaluronidase [7], consistent with the findings for our CA-MRSA isolate.

Unlike SCC*mec* types I–III, which carry multiple genes for various antibiotic resistance, SCC*mec* type IV only carries *mecA* conferring resistance to β-lactams. CA-MRSA strains are usually susceptible to tetracyclines, clindamycin, and TMP-SMX but not β-lactams [7]. These susceptibility patterns were also found in our CA-MRSA isolate. Thus, clindamycin, and TMP-SMX might be options for skin and soft-tissue infections. For complicated skin and soft-tissue infections and invasive MRSA infections caused by CA-MRSA, such as bloodstream infections, endocarditis, meningitis, and pneumonia, parenteral vancomycin is the recommended treatment. Linezolid might be an alternative agent for severe MRSA [29].

To our knowledge, this is the first report of a CA-MRSA isolate (spa type t13880) carrying SCC*mec* IV from patients in Thailand. Unfortunately, HA-MRSA strains have been reported in Thailand, most commonly ST239-SCC*mec* III type followed by ST5-SCC*mec* II [30,31]. CA-MRSA infection has been rarely documented. Only a study by Lulitanond et al. in 2013 isolated two MRSA strains (one each from pig farm workers and a pig) of ST9-SCC*mec* IX in the community [32]. SCC*mec* IX MRSA (belonging to spa type t337) was also reported by Larsen et al. in pigs in Thailand [33]. Thus, the emergence of SCC*mec* IX in animal farms in the community is concerning.

The reported CA-MRSA clones vary by region/country, including ST1-SCC*mec* IV (USA400) and ST8-SCC*mec* IV strains (USA300) in the United States, ST30-SCC*mec* IV and ST93-SCC*mec* IV strains in Australia, ST22-SCC*mec* IV strains in India, and CC80-SCC*mec* IV strains in Europe and Africa [34].

Interestingly, we classified our CA-MRSA isolate as spa type t13880 belonging to ST2885 by MLST analysis. This MRSA-ST288517 clone had only been identified in the Lao People’s Democratic Republic (bordering country of Thailand), but the ST2885 isolates belonged to spa type t13849. Additionally, these ST2885 isolates carried *eta* (encoding exfoliative toxin A), which was also found in our studied CA-MRSA isolate. However, most ST2885 strains are methicillin-sensitive *S. aureus* [35]. Therefore, the present study confirmed the existence of the ST2885 clone in Southeast Asia, albeit with a different methicillin susceptibility pattern.

Previously, Mekviwattanawong et al. determined the prevalence of infections caused by CA-MRSA among hospitalized patients, with MRSA isolated from the patients within 72 h of hospitalization. CA-MRSA was found in 2 of 186 patients with MRSA infection. The infections were likely caused by HA-MRSA because both patients had previous histories of hospital visits prior to their hospitalization. Moreover, the isolates defined as CA-MRSA strains were not subjected to SCC*mec* typing, and their antibiotic susceptibility profiles indicated multidrug resistance [9]. Thus, the situation of CA-MRSA in Thailand could not be confirmed until the present study. Treatment regimens for infections caused by *S. aureus* in the community setting should be developed.

## 5. Conclusions

We have isolated the ST2885-SCC*mec* IV CA-MRSA strain from a patient admitted to the hospital for the first time in Thailand. The presence of this isolate highlights the problem of methicillin-resistant infections in the community setting.

## Figures and Tables

**Table 1:** The identified antibiotic resistance genes in community acquired methicillin-resistant *Staphylococcus aureus* (CA-MRSA) no. S17.

Resistance Gene	Identity	Template Length	Contig	Position in Contig	Predicted Phenotype	Accession Number
**β-lactam**						
blaZ	100	849/849	NODE_15_length_14257_cov_356.776825	7028–7876	β-lactam resistance	JBTH01000015
mecA	100	2007/2007	NODE_1_length_202378_cov_183.284088	83,894–85,900	β-lactam resistance	BX571856
**Macrolide**						
Inu(A)	99.79	486/486	NODE_34_length_2491_cov_187.230835	2007–2492	Lincosamide resistance	M14039
**Tetracycline**						
tet(K)	99.86	1380/1380	NODE_6_length_4546_cov_373.607574	2335–3714	Tetracycline resistance	U38656
**Trimethoprim**						
dfrG	100	498/498	NODE_1_length_202378_cov_183.284088	90,645–91,142	Trimethoprim resistance	AB205645

**Table 2:** The identified genes of virulence factor in CA-MRSA no. S17.

Virulence Factor	Identity	Template Length	Contig	Position in Contig	Protein Function	Accession Number
*eta*	100	843/843	NODE_140_length_521104_cov_145.043243	517966..518808	Exfoliative toxin A	AP008953.1
*hlgA*	99.89	930/930	NODE_83_length_193242_cov_168.671219	110976..111905	Gamma-hemolysin chain II precursor	AP014942.1
*hlgB*	100	977/977	NODE_83_length_193242_cov_168.671219	113422..114398	Gamma-hemolysin component B precursor	AP014942.1
*hlgC*	99.79	948/948	NODE_83_length_193242_cov_168.671219	112473..113420	Gamma-hemolysin component C	AP014653.1
*lukD*	99.8	984/984	NODE_118_length_478021_cov_147.302170	469250..470233	Leukocidin D component	AP014653.1
*lukE*	99.89	936/936	NODE_118_length_478021_cov_147.302170	470235..471170	Leukocidin E component	BA000018.3
*lukE*	99.89	936/936	NODE_118_length_478021_cov_147.302170	470235..471170	Leukocidin E component	CP001781.1

## Data Availability

The data presented in this study are available in article.
